# Impact of pre-gestational and gestational diabetes mellitus on the expression of glucose transporters GLUT-1, GLUT-4 and GLUT-9 in human term placenta

**DOI:** 10.1007/s12020-016-1202-4

**Published:** 2016-12-16

**Authors:** Paweł Jan Stanirowski, Dariusz Szukiewicz, Michał Pyzlak, Nabil Abdalla, Włodzimierz Sawicki, Krzysztof Cendrowski

**Affiliations:** 1 Department of Obstetrics, Gynecology and Oncology, II Faculty of Medicine, Medical University of Warsaw, Mazovian Bródno Hospital, Kondratowicza 8, 03-242 Warsaw, Poland; 2 Department of General and Experimental Pathology with Centre for Preclinical Research and Technology (CEPT), II Faculty of Medicine, Medical University of Warsaw, Warsaw, Poland

**Keywords:** Glucose transporter, Placenta, Gestational diabetes mellitus, Pre-gestational diabetes mellitus, Quantitative morphometry

## Abstract

**Purpose:**

Various studies in placental tissue suggest that diabetes mellitus alters the expression of glucose transporter (GLUT) proteins, with insulin therapy being a possible modulatory factor. The aim of the present study was quantitative evaluation of the expression of glucose transporters (GLUT-1, GLUT-4, GLUT-9) in the placenta of women in both, uncomplicated and diabetic pregnancy. Additionally, the effect of insulin therapy on the expression of selected glucose transporter isoforms was analyzed.

**Methods:**

Term placental samples were obtained from healthy control (*n* = 25) and diabetic pregnancies, including diet-controlled gestational diabetes mellitus (GDMG1) (*n* = 16), insulin-controlled gestational diabetes mellitus (GDMG2) (*n* = 6), and pre-gestational diabetes mellitus (PGDM) (*n* = 6). Computer-assisted quantitative morphometry of stained placental sections was performed to determine the expression of selected glucose transporter proteins.

**Results:**

Morphometric analysis revealed a significant increase in the expression of GLUT-4 and GLUT-9 in insulin-dependent diabetic women (GDMG2 + PGDM) as compared to both, control and GDMG1 groups (*p* < .05). Significantly increased GLUT-1 expression was observed only in placental specimens from patients with PGDM (*p* < .05). No statistically significant differences in GLUT expression were found between GDMG1 patients and healthy controls.

**Conclusions:**

The results of the study confirmed the presence of GLUT-1, GLUT-4 and GLUT-9 proteins in the trophoblast from both, uncomplicated and diabetic pregnancies. In addition, insulin therapy may increase placental expression of GLUT-4 and GLUT-9, and partially GLUT-1, in women with GDMG2/PGDM.

## Introduction

In the course of pregnancy, transplacental nutrient transport of amino acids, lipids, and carbohydrates designed to meet the demands of the growing fetus constitutes the prerequisite condition of proper fetal development. Glucose, belonging to the last group, is the principal energy substrate for the fetus, the majority of which is supplied from the maternal circulation. Besides the difference in glucose concentration gradients in the maternal and fetal circulation, the intensity of placental metabolism and blood flow, the expression and activity of specific transporters is one of the key elements, which determine the ability of the human placenta to transfer glucose. As a consequence, for nearly three decades, transporter-dependent glucose flux across the placenta has been the subject of numerous studies, aiming to evaluate the expression of selected transporter isoforms and its effect on maternal-fetal glucose exchange [1–9]. Particular attention has been paid to changes in the expression and activity of glucose transporters in diabetic pregnancy, where disturbances in transplacental energy substrate supply may constitute one of the pathogenetic mechanisms leading to fetal overgrowth [10].

Members of the facilitative glucose transporter (GLUT) family vary in terms of substrate specificity, kinetics, localization and expression in human tissues, and are responsible for sodium-independent and concentration gradient-compliant hexose transfer [11, 12]. In terms of structure, all GLUT proteins encoded by the *SLC2A* gene family consist of ~500 amino acids and comprise 12 transmembrane domains connected by hydrophilic loops with both N and C termini localized in the cytoplasm [11–13]. Out of the identified 14 transporter isoforms, three (GLUT-1, GLUT-4 and GLUT-9) seem to be of particular importance for placental glucose exchange. GLUT-1, which is present in the majority of human tissues and, as a result, is considered to be a constitutive transporter isoform, in the placenta is found in the endothelial cells of placental villi, cyto and syncytiotrophoblast, at the same time being the primary transporter responsible for glucose transfer [1–5]. The characteristic for GLUT-4 close dependency on insulin stimulation results in an important role of the transporter in maintaining whole-body glucose homeostasis, whose presence was demonstrated together with insulin receptors in stromal cells of the placental villi [7]. The last of the transporters, GLUT-9, is the least known among all of the above mentioned isoforms, capable of glucose and fructose transfer, with positive expression in the placental syncytium and vascular endothelium [8, 9].

Both, gestational diabetes mellitus (GDM) and pre-gestational diabetes mellitus (PGDM) have been demonstrated to contribute to changes in the expression and activity of glucose transporters in the placenta, with insulin therapy as a possible modulatory factor [8, 14–17]. It has been speculated that increased glucose flux into the fetal circulation, followed by fetal hyperinsulinemia, increased production of the growth factors, and finally macrosomia, may be the consequences of these changes [14, 18]. The hypothesis, however, needs to be verified, taking into account the fact that the vast majority of the literature reports have been small sample-size or have focused on the expression of individual isoforms, mainly GLUT-1. Moreover, analysis of transporter expression only in membrane fractions of the trophoblast in the studies conducted so far may not have fully reflected the changes in the diabetic placenta taking into account intracellular localization of some GLUT isoforms, e.g. GLUT-4 or GLUT-1 [19, 20].

The aim of the present study was quantitative evaluation of the expression of GLUT-1, GLUT-4 and GLUT-9 glucose transporters in the placenta of women in both, healthy pregnancy and pregnancy complicated by GDM/PGDM. Additionally, the effect of insulin therapy on the expression of selected GLUT isoforms in the placenta of women with diabetes was analyzed.

## Material and methods

### Patients

The placental samples were obtained from 53 women after vaginal or cesarean delivery at the Department of Obstetrics, Gynecology and Oncology, II Faculty of Medicine, Medical University of Warsaw. Local Ethics Committee approved of the study and all participants signed their written informed consent (reference no. KB/150/2013). The inclusion criteria were as follows: maternal age >18, singleton pregnancy, and gestational age of >37 weeks. Fetal malformations, intrauterine fetal growth restriction, maternal chronic or pregnancy-induced hypertension, chronic renal or hepatic disease, in vitro fertilization, premature rupture of membranes, and smoking constituted the exclusion criteria.

The patients were divided into two groups: (i) study group comprising women with GDM or PGDM, and (ii) age-matched control group comprising women in an uncomplicated pregnancy. GDM was diagnosed based on the 75 g oral glucose tolerance test (OGTT) performed between 24 and 28 gestational weeks, in accordance with the criteria defined by the Hyperglycemia and Adverse Pregnancy Outcome study [21]. GDM patients received dietary advice at the initial stage and insulin therapy was introduced only in case of repeatedly inadequate glycemic control (fasting blood glucose level >90 mg/dl and/or 1-h postprandial blood glucose level >120 mg/dl). PGDM patients received insulin over the entire course of pregnancy. In the periconceptional period, all PGDM women remained under the care of the Diabetes Outpatient Clinic and only patients with good glycemic control, whose concentration of glycated hemoglobin within the 3 month period before pregnancy did not exceed 6%, were included. PGDM patients with preexisting medical conditions including cardiovascular diseases, nephro and retinopathy were excluded from the study.

Diabetic patients were classified according to the White’s Classification as White class A, gestational diabetes controlled with diet alone (GDMG1) or diet and insulin (GDMG2), White class B, pre-gestational diabetes mellitus with the onset at the age of ≥20, and White class C, PGDM with the onset at the age of 10–19. All patients from the study group were followed-up in the out-patient hospital clinic until 38 weeks of gestation.

### Placental tissue collection and immunohistochemical staining

Two separate cross-section specimens from the central and the peripheral region of the placenta were collected immediately upon vaginal delivery/cesarean section, fixed in a 10% buffered formalin and embedded in paraffin. After fixation, nine paraffin 5 μm sections (three for each GLUT isoform) were prepared for each of the examined placental specimens. Paraffin-embedded sections were stained using an IHC Select^®^ HRP/DAB kit from Merck Millipore (Darmstadt, Germany) and according to the protocol recommended by the manufacturer. Briefly, the slides were deparaffinized using xylene and rehydrated with ethanol. Preliminary antigen retrieval was achieved by high temperature processing of the samples in citrate buffer (pH 6.0) for 10 min. and endogenous peroxidase activity was quenched by incubation of the sections with 3% H_2_O_2_ for 20 min. The sections were subsequently incubated with blocking buffer (5% normal goat serum diluted in phosphate buffered saline, PBS), followed by overnight incubation with primary antibodies at 4 °C. Respective primary antibodies were used: (1) mouse monoclonal antibody to GLUT-1 (NBP1-35926, Novus Biologicals, USA, dilution 1:200), (2) rabbit polyclonal antibody to GLUT-4 (ab654, Abcam, USA, dilution 1:500), and (3) rabbit polyclonal antibody to GLUT-9 targeting both GLUT-9a and GLUT-9b variants (ab104623, dilution 1:1000). Following primary incubation, the sections were washed and incubated with horse radish peroxidase (HRP)-conjugated goat anti-mouse (NB7539, 0.5% v/v) or goat anti-rabbit (ab97051, 0.5% v/v) secondary IgG for 60 min. at room temperature. In order to visualize the specifically bound anti-GLUT primary antibodies, 3,3′-diaminobenzidine served as the chromogen for HRP, which generates a brown to black-colored polymeric oxidation product. Finally, the sections were counterstained with hematoxylin, dehydrated and mounted. Assuming the lack of cross-reactivity between pairs of GLUT isoforms (less than 35% of identities between GLUT-4 vs. GLUT-9, GLUT-1 vs. GLUT-4, and GLUT-1 vs. GLUT-9 using the BLAST technique), the respective negative controls for immunostainings were prepared simultaneously by replacing anti-GLUT-1, anti-GLUT-4, and anti-GLUT-9 with normal mouse or rabbit pre-immune IgG diluted with PBS at the same protein concentration as that used for the primary antibodies. Immunostained sections were examined and photographed using a Leica DMLB light microscope.

### Mean density of placental microvessels

Considering the fact that some GLUT are expressed in endothelial cells, it was presumed that the accuracy of GLUT expression measurement in the placental tissue may be influenced by the local differences in density of placental microvessels. To minimize this discrepancy in the results, identification of the vascular elements in the placental sections was performed using endothelial cells marker—rabbit polyclonal antibody against CD31 (ab28364, dilution 1:50). The tissue was probed with the primary antibody for 30 min. Subsequently, a HRP-conjugated goat anti-rabbit antibody was used as the secondary. Using light microscopy with computed morphometry for quantitative analysis (Quantimet 500C+ Image Processing and Analysis System, Leica Cambridge Ltd., Cambridge, United Kingdom), the vascular/extravascular tissular index (V/EVTI) was estimated in the calibrated areas of the placental sections, as described in detail elsewhere [22, 23]. Briefly, each paraffin section underwent three area analyses repeated by two independent observers. To minimize bias, within the calibrated areas of the placental sections, analyzed visual fields have been chosen randomly using the morphometric software. The single area measured with the picture analyzer amounted to 693287 μm^2^ and Table 1 provides the total number of placental specimens, sections and visual fields analyzed in the respective groups. The image analysis procedure consisted in the measurement of the total vascular area. Consequently, the total lumen area of all types of the identified vessels was summed up in diabetic and non-diabetic placentas. With the purpose of a minimizing disruption caused by technical errors, especially uniaxial section of the vessel, the lowest value of the Ferret’s diameter was accepted as the diameter of a single lumen. Thus, V/EVTI represents the ratio which reflects the intensity of vascularization and is most closely correlated with density of placental microvessels.

**Table 1:** Summary of the material collected in the study

Placental specimens	*N*	Central (A) peripheral (B)	GLUT-1 (sections × visual fields)	GLUT-4 (sections × visual fields)	GLUT-9 (sections × visual fields)
Group					
GDMG1	16	A: 16	48 × 3	48 × 3	48 × 3
		B: 16	48 × 3	48 × 3	48 × 3
GDMG2	6	A: 6	18 × 3	18 × 3	18 × 3
		B: 6	18 × 3	18 × 3	18 × 3
PGDM	6	A: 6	18 × 3	18 × 3	18 × 3
		B: 6	18 × 3	18 × 3	18 × 3
Control	25	A: 25	75 × 3	75 × 3	75 × 3
		B: 25	75 × 3	75 × 3	75 × 3
**Total: 53**		A: 53	159 × 3	159 × 3	159 × 3
		B: 53	159 × 3	159 × 3	159 × 3
		**A + B = 106**	**A + B = 954 × 3 = 2862 images**		

Specimens obtained from central (A) and peripheral (B) regions of the placenta in the respective groups of diabetic patients (*GDMG1* diet-controlled gestational diabetes mellitus, *GDMG2* insulin-controlled gestational diabetes mellitus, *PGDM* pregestational diabetes mellitus) and non-diabetic controls. Three sections have been prepared from each placental specimen for each of the analyzed GLUT: GLUT-1, GLUT-4, GLUT-9. Next, from each section a digital camera captured the images of three visual fields in the optical system

### Quantitative morphometric analysis of GLUT expression

Following immunostainings, a quantitative immunohistochemistry based on the morphometric software (Quantimet 500C+) was applied for GLUT-1, GLUT-4 and GLUT-9 identification in placental sections under light microscopy. All morphometric procedures were carried out twice by two independent researchers and the average values were uploaded in the result recording tab. Intensity of immunostaining was evaluated using mean color saturation parameter and thresholding in grey-level histograms. Thus, expression of the respective GLUT corresponded to the total immunostained calibrated area of the examined sections, where color saturation comprises segmentation-separation criteria for the objects. A single analyzed image area amounted to 134755 μm^2^ (magnification 400x). In order to achieve a maximum accuracy of the measurements, the following factors have been controlled or monitored: averaging of image intake, hue, illumination, luminance, power supply, relation of illumination to quantification of the area percentage of the positively staining structures, shading correction, and warming up. More detailed description of these morphometric procedures was given previously elsewhere [23]. During comparative measurements of GLUT expression in placental tissue samples in the studied groups, the vascular density-matched samples were analyzed. In any case, the difference between median V/EVTI values did not exceed ±5%.

### Statistical analysis

Statistical analysis was performed using the R package v.3.2.1 (The R Foundation for Statistical Computing, Vienna, Austria). Continuous variables were compared using the Kruskal–Wallis rank sum test with the post-hoc Dunn’s test and for categorical variables the chi-square test with the Bonferroni correction was applied. The results were expressed as median and interquartile range (IQR), or median of percentage values and IQR. The *p*-value of <0.05 was considered as statistically significant.

To assess the inter and intra-observer agreement in immunohistochemical images, interpretation kappa statistic (*ĸ*) was used. The observer agreement was considered almost perfect when *ĸ* value was above 0.80; a *ĸ* value of 0.61–0.80, substantial agreement; a *ĸ* value of 0.41–0.60, moderate agreement; a *ĸ* value of 0.21–0.40, fair agreement; and a *ĸ* value of less than 0.20 indicated poor agreement.

## Results

After the completion of data collection, 25 women in uncomplicated pregnancy were assigned to the control group, while 28 age-matched patients constituted the study group comprising of 16 GDMG1, 6 GDMG2, and 6 PGDM type 1 (2 White class B and 4 White class C) diabetics. The characteristics of the patients from whom placental samples were obtained are shown in Table 2. Statistically significant differences between the subgroups of patients with GDM and non-diabetic controls were found in fasting glucose (*p* < .05), as well as 1-h and 2-h plasma glucose concentrations during OGTT (*p* < .01). With regard to the perinatal data, the analysis revealed that both placental and fetal weights in the diet-controlled GDM group were significantly lower as compared to other diabetic patients and with an uncomplicated pregnancy (*p* < .05). Fetal macrosomia proved to be an additional, statistically significant differentiation factor between PGDM and diet-controlled GDM patients (*p* < .05), and with a two-fold higher incidence as compared to controls (*p* = .08). Mean third trimester concentrations of glycated hemoglobin in the GDMG1, GDMG2 and PGDM groups were 5.23 ± 0.34%, 5.87 ± 0.37% and 5.68 ± 0.66%, respectively (normal range: ≤6%).

**Table 2:** Patient and pregnancy characteristics in diabetic and control populations

	GDMG1 (*n*=16)	GDMG2(*n*=6)	PGDM (*n*=6)	Control (*n*=25)	*p* value
Age (years)	33 [30–37]	34 [29–37]	35 [33–36]	30 [28–32]	.10
Gestational age (weeks)	39 [38–39]	39 [38–39]	37 [37–38]	39 [39–39]	.07
Gravidity	2 [2–3]	2 [2–3]	1 [1–1]	2 [1–2]	.11
Parity	2 [1–2]	2 [1–2]	1 [1–1]	2 [1–2]	.13
Pre-pregnancy BMI (kg/m^2^)	25.9 [22.4–27.1]	23.2 [22.1–32.2]	22.1 [19.3–23.1]	22.6 [21.8–24.1]	.14
Fasting plasma glucose (mg/dl)^a^	86 [80–97]	98 [96–112]	–	79 [74–83]	<.05^*^
1-h plasma glucose (mg/dl)^a^	181 [159–186]	162 [148–188]	–	120 [102-144]	<.01^*^
2-h plasma glucose (mg/dl)^a^	156 [138–163]	153 [143–158]	–	103.5 [85.5–116.5]	<.01^*^
Mode of delivery					
Vaginal delivery	6 (37.5%)	0	0	9 (36.0%)	.11
Cesarean section	10 (62.5%)	6 (100%)	6 (100%)	16 (64.0%)	
Fetal weight (g)	3270 [3180–3430]	3550 [2980–4410]	4010 [3790–4080]	3470 [3240–3810]	<.05^†^
Fetal sex					
Male	9 (56.3%)	2 (33.3%)	5 (83.3%)	14 (56.0%)	.63
Female	7 (43.7%)	4 (66.7%)	1 (16.7%)	11 (44.0%)	
Fetal macrosomia^b^	0	2 (33.3%)	3 (50.0%)	5 (20.0%)	<.01^‡^
Apgar score	10 [10–10]	10 [10–10]	10 [10–10]	10 [10–10]	.96
Placental weight (g)	523 [457–677]	634 [472–683]	733 [700–796]	597 [498–664]	<.05^†^
Fetal/ placental ratio	6.9 [5.1–7.2]	6.1 [5.3–7.1]	5.4 [5.4–5.6]	6.3 [5.5–6.7]	.24

Data are expressed as median [*IQR*, interquartile range], or as frequency (%)

*GDMG1* diet-controlled gestational diabetes mellitus, *GDMG2* insulin-controlled gestational diabetes mellitus, *PGDM* pre-gestational diabetes mellitus

^*^GDMG1, GDMG2 vs. control

^†^GDMG1 vs. GDMG2, PGDM, control

^‡^GDMG1 vs. PGDM

^a^results of the 75 g OGTT performed between 24–28 gestational weeks

^b^fetal macrosomia defined as birth-weight over 4000 g irrespective of gestational age

**Fig. 1:**
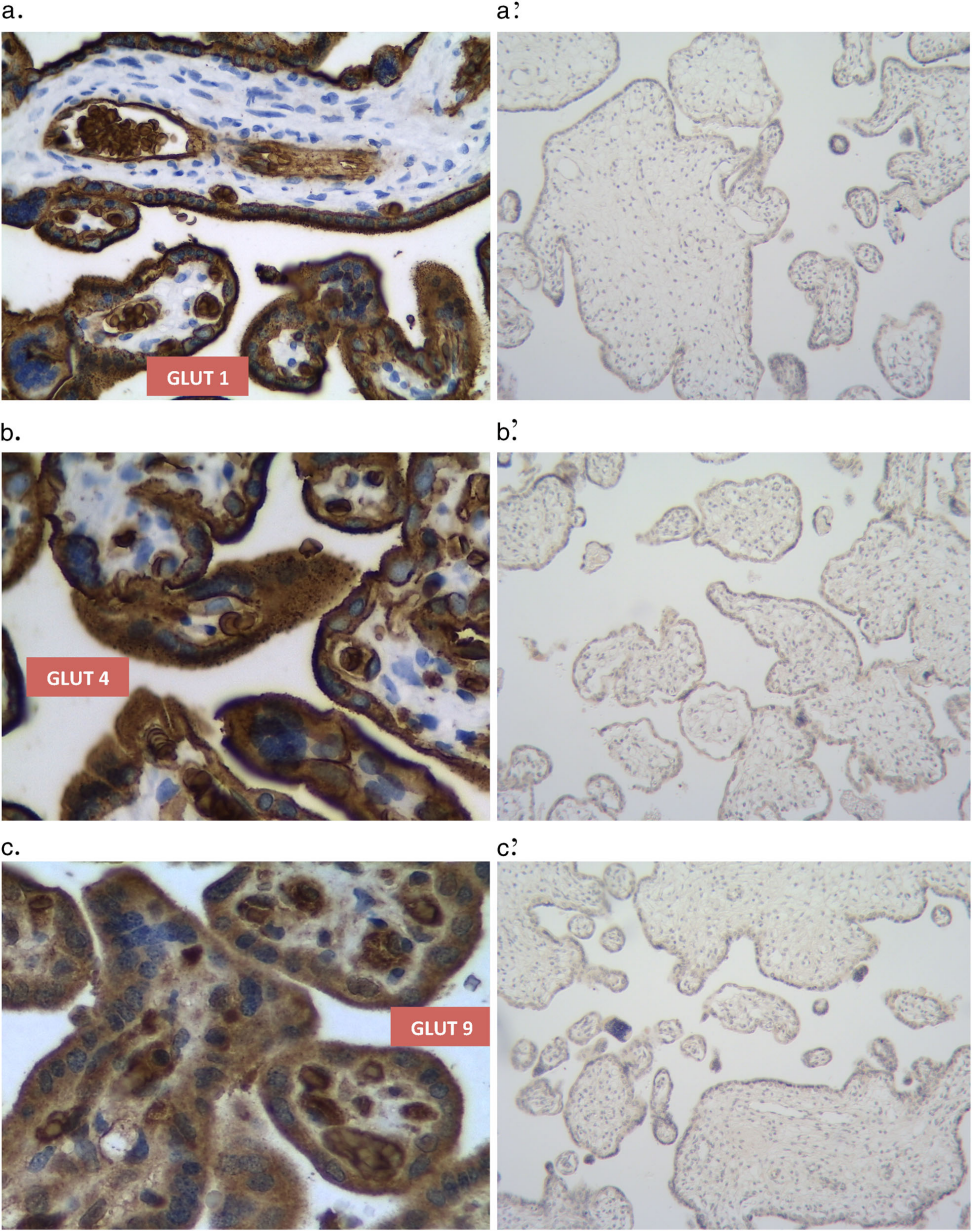
Immunohistochemical localization of GLUT in human term placenta at 400x magnification: GLUT-1 (**a**); GLUT-4 (**b**); GLUT-9 (**c**). The respective negative controls (**a**’), (**b**’), and (**c**’) are also presented (magnification 100×). A single visual field observed with the naked eye looks substantially uniformly within each studied group. For that reason, to avoid unjustified overloading with the pictures, only one immunostaining is presented for GLUT-1, GLUT-4, and GLUT-9

Immunohistochemical techniques used for identification of GLUT-1, GLUT-4 and GLUT-9 revealed the presence of all GLUT in the placental tissue (Fig. 1). Membrane expression of the immunostained protein in the apical region of the trophoblast, including the microvilli, corresponded predominantly with the GLUT-1 isoform, while the expressions of both, GLUT-4 and GLUT-9 were more blurry, shifted somewhat towards the cytoplasmic compartment of the trophoblast cells. None of the immunoreactions described above were observed when normal mouse or rabbit pre-immune IgG were used (Fig. 1).

**Fig. 2:**
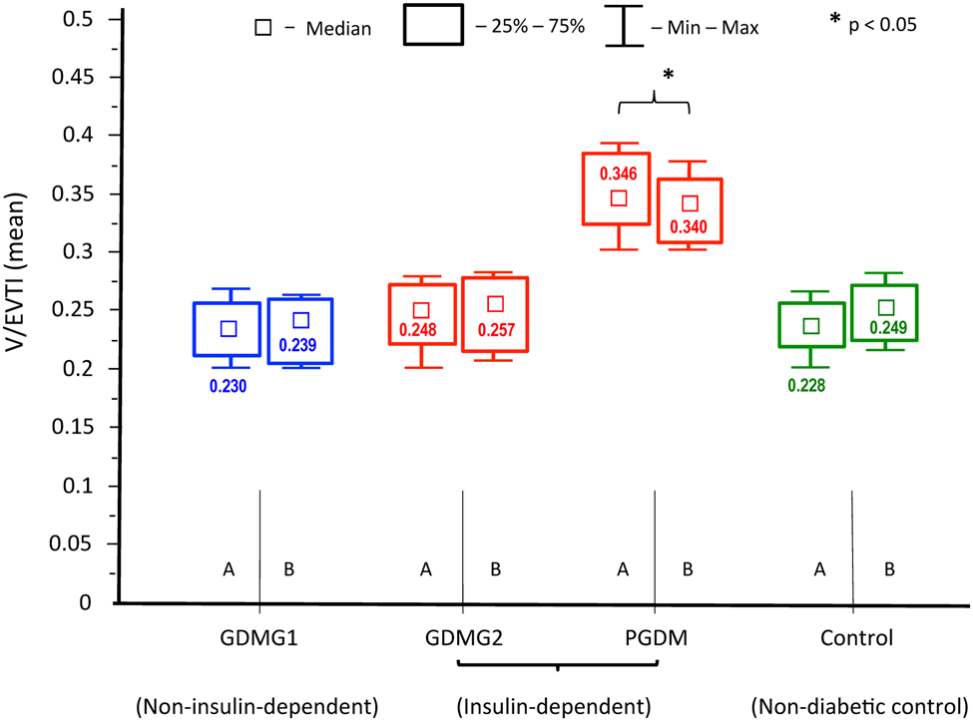
A comparative examination of microvessel density in the placental sections (A—central part, B—peripheral part of the placenta) using the vascular/extravascular tissular index (V/EVTI); the median values (abstract numbers) and IQR. *GDMG1* diet-controlled gestational diabetes mellitus, *GDMG2*, insulin-controlled gestational diabetes mellitus, *PGDM* pregestational diabetes mellitus

In both, the control and the study groups (except for PGDM), median V/EVTI values were increased in the specimens obtained from the peripheral region of the placenta as compared to the specimens from the central part, however the differences did not reach statistical significance (Fig. 2). As a result, further analyses of both V/EVTI and the respective GLUTs expression were performed altogether (specimens: A + B) within the diabetic and non-diabetic groups. The analysis revealed median V/EVTI values to be significantly increased in the placentas from PGDM patients as compared to the other groups (*p* < .05) (Fig. 2).

**Fig. 3:**
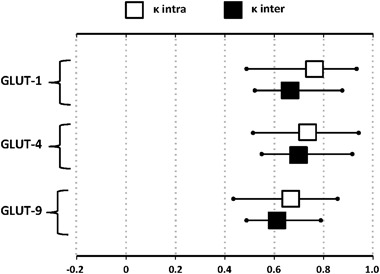
Intra and inter-observer agreement calculated for each studied GLUT isoform, measured with 95% confidence interval. The observer agreement was considered good (substantial to almost perfect) when the *ĸ* value was above 0.60

**Fig. 4:**
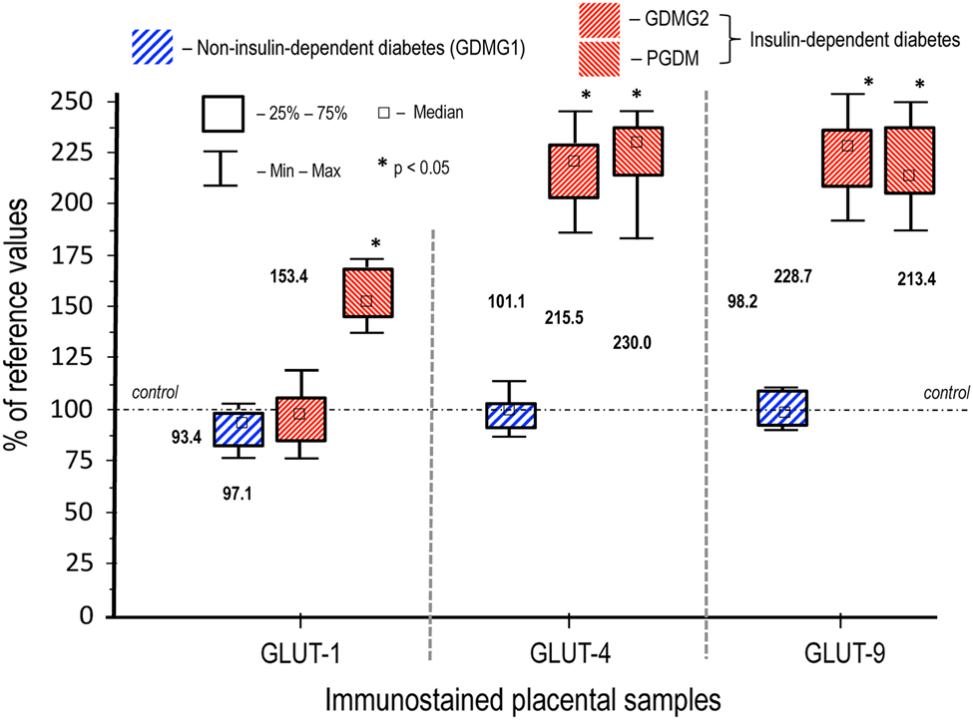
Expression of the respective glucose transporters (GLUT-1, GLUT-2, GLUT-9) in placental sections: diabetes-complicated pregnancy (non-insulin-dependent [GDMG1—diet-controlled gestational diabetes mellitus] and insulin-dependent [GDMG2—insulin-controlled gestational diabetes mellitus; PGDM—pregestational diabetes mellitus]) vs. vascular density-matched non-diabetic controls. Median of the percent values and IQR. The median value in the respective controls was taken as 100%*. * The value of the median in all controls (GLUT-1, *N* = 25; GLUT-4, *N* = 25; and GLUT-9, *N* = 25) have showed strong coherence with the respective mean value and the differences between the median and the mean under no circumstances did not exceeded ±5%

The results of the quantitative morphometric analysis performed for the vascular density-matched placental samples revealed that the intra-observer agreements were superior to the inter-observer agreements and the *ĸ* values for GLUT-1, GLUT-4, and GLUT-9 are summarized in Fig. 3. A significant increase in the expression of GLUT-4 and GLUT-9 in insulin-dependent diabetic patients (GDMG2 + PGDM) as compared to both, control and GDMG1 groups was noted, (*p* < .05; Fig. 4). Contrary to the two above-mentioned isoforms, significantly increased GLUT-1 expression was observed only in placental specimens from patients with PGDM (*p* < .05). No statistically significant differences in GLUT expression were found between GDMG1 patients and healthy controls.

## Discussion

The present study investigated the expression of GLUT, GLUT-1, GLUT-4 and GLUT-9, in stained placental sections from physiological and diabetes-complicated pregnancies using computer-assisted quantitative morphometry. To the best of our knowledge, this has been the first study to use the abovementioned method to evaluate the expression of selected GLUT isoforms in human term placenta. The results of immunohistochemical staining confirmed the presence of all GLUT proteins in the trophoblast from both, uncomplicated and diabetic pregnancies. Additionally, insulin therapy might be responsible for the increased placental expression of GLUT-4 and GLUT-9, and partially GLUT-1, in women with GDMG2/PGDM.

GLUT-1 is the most common GLUT found in the human placenta. Thus, it is believed to be the main isoform responsible for glucose transfer into the fetal circulation by the vast majority of researchers [1, 2, 4, 19]. In the syncytium, GLUT-1 expression is asymmetric, with three-fold higher density in the maternal-facing microvillous membrane (MVM) as compared to the fetal-facing basal membrane (BM) [2]. In the present study, transporter expression was the most intense in the trophoblast membranes, including microvilli, which is consistent with previous reports [1–5, 19]. Contrary to transporter localization, attempts at quantitative analysis of GLUT-1 expression in the placenta have so far failed to generate unambiguous results. Studies using membrane fractions of the syncytium either found no differences between transporter expression in MVM and BM of GDM women and healthy controls, or reported increased expression only in the BM fraction [14, 16]. On the other hand, recent use of unfractionated trophoblast membranes has revealed a significant increase of the GLUT-1 expression only in insulin-controlled GDM patients [17]. In the present study, morphometric analysis demonstrated no differences in GLUT-1 expression between GDM women and healthy controls, similarly to the results of Jansson et al. [16]. The reasons for the discrepancies between the remaining authors remains unclear. However, the analysis of the GLUT-1 expression in separate tissue fractions of the trophoblast, as well as different diagnostic criteria and thresholds of glycemic control in GDM patients, should be taken into consideration. In case of PGDM women, results of the earlier studies demonstrated higher GLUT-1 expression in syncytial BM, without significant differences with regard to MVM, which to some extent confirms the general increase in transporter density observed in morphometry [14, 15].

We have demonstrated a significant increase in the GLUT-9 expression in the placental samples from GDMG2 and PGDM women, as well as lack of differences when GDMG1 patients and healthy controls were compared. Similar results were reported by the authors of the only study, so far, to investigate the expression of two separate GLUT-9 isoforms: GLUT-9a and GLUT-9b in membrane fractions of the syncytiotrophoblast in diabetic placentas [8]. As for GLUT-9a, the authors found significantly increased expression in syncytial BM from the diabetic pregnancies, including GDMG1, whereas GLUT-9b was predominant in MVM and BM only in GDMG2/PGDM women. In light of the fact that the present study did not analyze the expression of the individual GLUT-9 variants, the results of Bibee et al., do not differ notably from our quantitative morphometric findings. Taking into account the main objectives, the results from both studies confirmed that GLUT-9 is involved in maternal-fetal hexose exchange, and that trophoblastic expression of the transporter is significantly increased in diabetic women, especially during insulin therapy. Importantly, the fact that GLUT-9 belongs to class II of the facilitative transporter family is the reason why its ability to transfer fructose, whose excess—as in the case of glucose—may be detrimental to fetal development, constitutes one of its most significant properties. Patients with diabetes are known to have elevated fructose levels in the blood, and fructose concentrations in the umbilical cord and fetal blood are higher as compared to maternal levels [24, 25]. The latter may be both, a result of the endogenous production of fructose by the fetal-placental unit in physiological pregnancy using glucose as the substrate, or—as was proven by animal studies—a response to excessive maternal fructose intake [25, 26]. Vickers et al., who administered fructose solution to pregnant rats in order to provide 20% of the daily caloric intake from fructose, reported maternal hyperinsulinemia and significantly higher birth weight in the offspring [26]. One of the possible explanations might be an increased GLUT-9 expression in the placenta, however studies on transplacental fructose transport and its effect on the development of human fetus are lacking and the only results are based on animal models. In this respect, it should be noted that the expression of other fructose transporters, including the primary GLUT-5, has not been confirmed in the placenta so far [11, 18].

Unlike the two mentioned GLUT, in the initial studies on the presence of GLUT-4 in the placenta it was found to be absent or barely detectable [1, 4, 6]. It was not until the study by Xing et al., that presence of GLUT-4 protein was finally confirmed in stromal cells of the placental villi [7]. The trophoblastic expression of GLUT-4 in human term placenta reported in our study is an important finding regarding the possibilities of regulating transplacental glucose transfer and the effect of insulin on the intensification of intrauterine fetal growth. It has been well-documented that the expression of GLUT-4 in human tissues, e.g. adipose tissue or muscles, is closely correlated with insulin stimulation, whose receptors can be found, among others, in the placental syncytium [11, 27, 28]. In the course of insulin therapy, increased GLUT-4 expression in the trophoblast may be observed in women with GDMG2/PGDM, as was confirmed by the results of quantitative morphometry. Direct consequences might include increased glucose flux into the fetal circulation, intensified fetal growth and the subsequent macrosomia, which in the present study was more common in the insulin-dependent diabetic groups. Nonetheless, the presented hypothesis ought to be treated with caution, taking into account results of the studies which found no or only transient stimulatory effect of insulin on glucose transfer in human term placenta [19, 29–31]. In addition, the findings of three different studies which compared the expression of GLUT-4 with clinical data not only found no differences but also detected a decrease in the placental transporter expression in women with fetal macrosomia and/or insulin-dependent diabetes [6, 7, 17]. One must remember, however, that the number of insulin receptors in the syncytium decreases as pregnancy progresses, and the abovementioned studies evaluated GLUT-4 expression only in the membrane fractions of the trophoblast, while the majority of transporter is found in the intracellular compartment [11, 27].

The analysis of the subgroups revealed a significantly higher incidence of macrosomia among women with PGDM as compared to diet-controlled GDM. Also, the rate of macrosomia in the former group was two-fold higher as compared to healthy controls. These findings are consistent with the increased expression of GLUT-4 and GLUT-9 transporters, and also partly GLUT-1, in the placentas of women with insulin-dependent diabetes. The lack of cases of fetal overgrowth in the GDMG1 group in the present study might be the result of strict dietary adherence, which is confirmed by normal values of third trimester glycated hemoglobin concentrations, and similar expression of GLUT isoforms as compared to healthy controls. On the other hand, the fact that 20% of the fetuses born to healthy mothers were macrosomic indicates that glucose transfer is not the only determinant of fetal growth and/or that there are differences in the mechanisms regulating glucose transfer in diabetic placentas as compared to physiological pregnancies. For example, in the first case, the enhanced lipoprotein lipase activity and expression of fatty acid binding proteins in the placenta of diabetic women may account for the excessive transfer of free fatty acids into the fetal circulation and subsequent fetal overgrowth [32]. On the other hand, the latter assumption is supported by the study of Osmond et al., who demonstrated a decreased uptake and transfer of glucose in perfused placentas from diet-controlled GDM women as compared to healthy controls and insulin-controlled GDM women [31]. In light of the abovementioned data and our findings, it seems safe to conclude that, in the initial stages of GDM, elevated levels of glucose trigger processes which limit the transfer of glucose from the maternal to fetal circulation without a significant change in the total number of transporters. Post-translational modification of the transporters, recently postulated by Acosta et al., or their intracellular translocation within the trophoblast observed in vitro, are examples of such processes [9, 20]. As a result, the fetus is protected from the detrimental effect of maternal hyperglycemia, and the consequent threat of macrosomia. Adequate glycemic control is the necessary prerequisite in maintaining that condition and if the insulin therapy needs to be initiated, the protective mechanisms are attenuated, leading to excessive transfer of glucose to the fetus. The finding that glycated hemoglobin levels were within the normal range in the majority of insulin-dependent women in the peripartum period and fetal macrosomia still occurred in 33–50% of them indicates that insulin may be one of the main factors which promote increased GLUT expression and fetal overgrowth even under strict glycemic control.

Both the placental and the fetal weights in an uncomplicated pregnancy were significantly higher compared to the GDMG1 patients, which, as there were no differences in GLUT expression, may suggest that it is the larger exchange surface area of the placenta and consequently the larger total number of transporters rather than their density that affects the birthweight of the fetus [6, 9]. The above assumption could be of importance, especially in cases of well-controlled GDMG1 and, apart from the already specified mechanisms, could provide further explanation of our observations. In the case of GDMG2/PGDM patients, the insulin may simultaneously stimulate placental growth and increase in the expression of GLUT, leading to more intensive fetal growth. However, as there are no studies evaluating the correlation of GLUT expression with measurements of the maternal surface area of the placenta, we cannot definitely confirm the hypothesis.

The present study is subject to some limitations; for example, the fact that the method used assesses GLUT expression in the membranes as well as in the cytosol of the trophoblast, whereas only the former group is directly responsible for glucose transfer, may be questioned. One must remember, however, that the omission of the intracellular GLUT fraction in the quantitative analysis may not fully reflect the transport capacity of the trophoblast, particularly taking into account that this pool of transporters undergoes increased translocation towards the cell membranes in response to stimulating factors such as insulin [28, 33]. Another limitation of the study concerns the possible impact of the mode of delivery on GLUT protein expression considering there were no vaginal deliveries in the GDMG2/PGDM group. However, in the light of the studies evaluating GLUT-1 expression in MVM and BM of the syncytiotrophoblast, where no differences were demonstrated in the transporter density between vaginal deliveries and those by cesarean section, it seems unlikely that these factors are interdependent [2, 34].

In conclusion, the present study demonstrated the presence of GLUT-1, GLUT-4 and GLUT-9 in human term placenta, as well as an increased expression of GLUT in the placentas from insulin-dependent diabetic pregnancies. Also, the fact that macrosomia was more common in the group of women with insulin-dependent diabetes confirms the earlier assumption that excess glucose transfer across the placenta may be responsible for fetal overgrowth. Further studies aiming to identify the factors responsible for the increased number of GLUT transporters and the mechanisms which regulate transport of glucose in human diabetic placenta are therefore necessary.
